# Genome-scale computational analysis of DNA curvature and repeats in Arabidopsis and rice uncovers plant-specific genomic properties

**DOI:** 10.1186/1471-2164-12-214

**Published:** 2011-05-06

**Authors:** Ali Masoudi-Nejad, Sara Movahedi, Ruy Jáuregui

**Affiliations:** 1 Laboratory of Systems Biology and Bioinformatics (LBB), Institute of Biochemistry and Biophysics and COE in Biomathematics, University of Tehran, Tehran, Iran; 2 VIB Department of Plant Systems Biology, Ghent University, Gent, Belgium; 3 Microbial Interactions and Processes Research Group, Department of Medical Microbiology, Helmholtz Center for Infection Research, Braunschweig, Germany

## Abstract

**Background:**

Due to its overarching role in genome function, sequence-dependent DNA curvature continues to attract great attention. The DNA double helix is not a rigid cylinder, but presents both curvature and flexibility in different regions, depending on the sequence. More in depth knowledge of the various orders of complexity of genomic DNA structure has allowed the design of sophisticated bioinformatics tools for its analysis and manipulation, which, in turn, have yielded a better understanding of the genome itself. Curved DNA is involved in many biologically important processes, such as transcription initiation and termination, recombination, DNA replication, and nucleosome positioning. CpG islands and tandem repeats also play significant roles in the dynamics and evolution of genomes.

**Results:**

In this study, we analyzed the relationship between these three structural features within rice (*Oryza sativa*) and Arabidopsis (*Arabidopsis thaliana*) genomes. A genome-scale prediction of curvature distribution in rice and Arabidopsis indicated that most of the chromosomes of both genomes have maximal chromosomal DNA curvature adjacent to the centromeric region. By analyzing tandem repeats across the genome, we found that frequencies of repeats are higher in regions adjacent to those with high curvature value. Further analysis of CpG islands shows a clear interdependence between curvature value, repeat frequencies and CpG islands. Each CpG island appears in a local minimal curvature region, and CpG islands usually do not appear in the centromere or regions with high repeat frequency. A statistical evaluation demonstrates the significance and non-randomness of these features.

**Conclusions:**

This study represents the first systematic genome-scale analysis of DNA curvature, CpG islands and tandem repeats at the DNA sequence level in plant genomes, and finds that not all of the chromosomes in plants follow the same rules common to other eukaryote organisms, suggesting that some of these genomic properties might be considered as specific to plants.

## Background

The higher-order structure of DNA, including hairpin turns, bending and curvature, and precise chromatin topology, could provide novel metadata needed to explain genome complexity. The overall effect of electromagnetic interactions in the DNA molecule, described by various topological variables such as twist, slide, tilt and roll [1], is to deviate the trajectory of the DNA molecule from an ideal straight line to a curved one in some cases, depending on the sequence. Trifonov and Sussman [2] observed that the natural anisotropy of the DNA molecule facilitates its smooth folding into chromatin, and proposed the initial concept that certain DNA regions may be bent, especially in A-rich tracts. The curvature of DNA represents the tendency of the helix axis to follow a non-linear pathway over a substantial length. The pioneering work of Gabrielian et al. [3], Bolshoy [4] and Shpigelman et al. [5] demonstrated that every organism has a characteristic DNA curvature profile. Additional studies presented significant relationships between curvature and other factors such as centromeric sequence, amino acid composition and transcription regulation. For instance, all sixteen centromeres of *Saccharomyces cerevisiae *are curved [6], and the centromere sequence *par*C of *Escherichia coli *is strongly curved [7]; the codon usage and the aminoacid composition of the proteome are correlated with the DNA curvature profile [8,9], and discrete DNA curvature signals are conserved in regulatory regions of both eukaryote and prokaryote genes [10-12]. Recent data also shows that promoter regions are significantly more curved than coding regions or randomly permuted sequences [13]. It has also been recently hypothesized that DNA curvature could affect transcription termination in many prokaryotes either directly, through contacts with RNA polymerase, or indirectly, via contacts with some regulatory proteins [14]. A deeper understanding of the various orders of complexity of genomic DNA structure has allowed the design of sophisticated biochemical and biophysical tools for its analysis and manipulation, which in turn, have yielded a better knowledge of the genome itself. It has been shown that the inclusion of DNA structural parameters in the analysis of genomic properties leads to a better understanding of the underlying mechanisms regulating various biological functions [15].

Various programs to calculate DNA bending and curvature have been proposed since the initial description of the structural variables involved [16]. In parallel, different models compiling the contributions of DNA structural parameters have been made and compared, including A-tract based, dinucleotide, and trinucleotide models [17]. In more recent times structural algorithms that predict the DNA trajectory in 3D have been published and tested [18,19]. Furthermore, novel resources compiling reported structural parameter sets of the DNA molecule, and facilitating their analysis have been recently published [20,21]. The program "CURVATURE" [5] was among the first to allow the calculation of DNA curvature of an arbitrarily long DNA sequence, and provided a set of wedge angles as structural parameters estimated from experimental data. It has since then been tested and validated in numerous publications, making it the optimal choice for our analysis.

Comparative genome analyses have shown the existence of conserved gene orders (colinearity) in the genomes of different plant and mammal species [22]. Rice (a monocot from the grass family) and Arabidopsis (a dicot from the mustard family) are model monocot and dicot genomes that have been fully sequenced [23,24]. Comparison of the rice and Arabidopsis genomes and proteomes showed that 71% of predicted rice proteins were similar to Arabidopsis proteins. This promising and unexpected high similarity suggests that the cellular and biochemical functions of many rice genes can be interpreted through experiments conducted in Arabidopsis. Yet, further analysis is needed to clarify the relationship between these two plants, which belong to two different classes. CpG Islands are clusters of CpG dinucleotides in GC-rich regions, usually ~1 kb long [1]. They have been identified in the promoter regions of approximately 50% of genes in different organisms and are considered as gene markers. In 1987, Gardiner-Garden and Frommer [25] first proposed an algorithm for scanning CpG Islands in a DNA sequence, however, this algorithm significantly inflates the number of CpG Islands because of the many repeats which are abundant in plant genomes. To solve this problem, Takai and Jones [26] performed a systematic evaluation of the three parameters in Gardiner-Garden and Frommer's algorithm and provided an optimal set of parameters.

Tandem repeats are ubiquitous sequence features in both prokaryotic and eukaryotic genomes. A direct or tandem repeat is the same pattern recurring on the same strand in the same nucleotide order. Tandem repeats play significant structural and functional roles in DNA. They occur in abundance in structural areas such as telomeres, centromeres and histone binding regions [27]. It has been suggested that the conserved 3' region of some types of centromere-specific repeats have significant potential to direct bending [28,29]. These repeats also play a regulatory role when found near genes and perhaps even within genes. Short tandem repeats are used as a convenient tool for the genetic profiling of individuals or for genetic marker analysis in mapping studies. Thus, identification and analysis of repetitive DNA is an active area of biological and computational research. However, to the best of our knowledge, attempts have yet to be made to establish genome-scale relationships between DNA curvature, CpG islands, tandem repeats and centromeric regions of any organism. Therefore, we conducted a comparative genome-scale analysis of the Arabidopsis and rice genomes to identify possible relationships between their genomic curvature, CpG islands, tandem repeats and each chromosome's centromere, and additionally explored their biological significance.

## Results and discussion

The main objectives of repetitive pattern identification algorithms are to identify its periodicity, pattern structure, location and copy number. The algorithmic challenges for the repeat pattern identification problem are lack of prior knowledge regarding the composition of the repeat pattern and presence of inexact and hidden repeats. Inexact repeats are formed due to mutations of exact repeats and are thought to be representations of historical events associated with sequence evolution. Thus, it is important for any repetitive pattern identification algorithm to identify inexact in addition to exact repeat structures in a DNA sequence.

By applying the algorithms and programs described in the Methods section, we obtained nine plots for each of the chromosomes in rice and Arabidopsis genomes. This set of graphical plots provided the opportunity to study the relationships between the three major factors, curvature, CpG islands, and tandem repeats in relation to each other. These plots are:

• An average curvature plot along the whole chromosome

• Two plots for position and length of CpG islands

• Two Tandem Repeats plots; number of repeats (repetition plot) and length of repeats (length plot)

• Combined-plot of curvature and length of the CpG islands

• Combined-plot of curvature and repeats

• Combined-plot of repeats and CpG islands

• Combined-plot of curvature, repeats and CpG islands

### Curvature landscape

**Figure 1:**
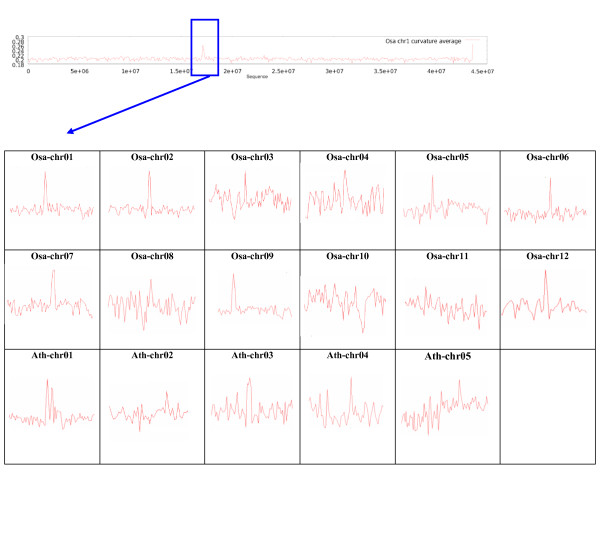
**Signal of average curvature value for rice and Arabidopsis chromosomes**. Curvature profile for rice chromosome 1 (top). Profiles around the centromeric region for 12 rice chromosomes and 5 Arabidopsis chromosomes (bottom). Maximal curvature values were observed for most of the chromosomes around the physical centromere location.

Figure 1 shows the average curvature values for all Arabidopsis and rice chromosomes. One of the most important features of these graphs is that in most of the chromosomes the centromeric regions are significantly curved and surprisingly the maximal curvature values (MaxCV) for these chromosomes are located in the same neighborhood as centromeric physical positions (CPP). Chromosomes 10 and 11 in rice and chromosomes 2 and 5 in Arabidopsis show MaxCV and CPP in different locations (for a complete curvature landscape of the genomes see additional file 1 "plant-plots"). Table 1 shows the centromeric physical position and the position of the maximal curvature value in rice and Arabidopsis chromosomes. In the Arabidopsis genome, the position of maximal curvature values of chromosomes 1, 3 and 4 occurs in the same range of CPP (60%). A similar pattern was observed for rice, in which, with the exception of chromosomes 10 and 11, the rest of the chromosomes follow this rule (83%). Since centromeres play a fundamental role during cell division and chromosome segregation, it is not far fetched to suppose that the curvature regions adjacent to the centromeres are also relevant for this process; however, this must be the subject of further studies.

**Table 1:** Approximate centromere and maximal curvature locations.

Chromosome	MaxCV	CPP	Difference
Osa_chr01	17100000	16800000	300000
Osa_chr02	13900000	13700000	200000
Osa_chr03	19900000	19500000	400000
Osa_chr04	9900000	9700000	200000
Osa_chr05	12600000	12400000	200000
Osa_chr06	15800000	15400000	400000
Osa_chr07	12500000	12200000	300000
Osa_chr08	13200000	12900000	300000
Osa_chr09	2900000	2700000	200000
Osa_chr10	**10600000**	**7700000**	**2900000**
Osa_chr11	**18300000**	**12000000**	**6300000**
Osa_chr12	12300000	12000000	300000
			
Ath_chr01	15000000	15089187	89167
Ath_chr02	**1200000**	**3608427**	**2408427**
Ath_chr03	14100000	13592000	508000
Ath_chr04	4200000	3956519	243481
Ath_chr05	**14100000**	**12742755**	**1357245**

The position of maximal curvature value (MaxCV) and centromeric physical position (CPP) in rice (Osa) and Arabidopsis (Ath) chromosomes are shown together with the difference between these two positions. Chromosomes with exceptional features are in bold. Note that these numbers are the center of a physical range between 200,000-500,000 bp.

### Survey of tandem repeats and CpG islands

**Figure 2:**
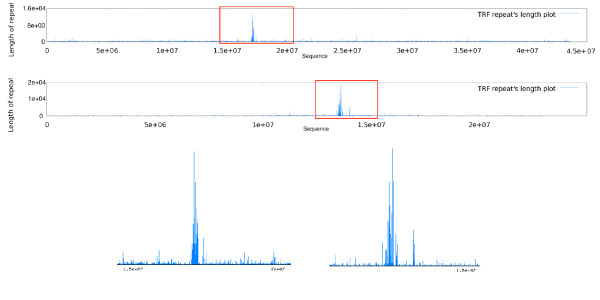
**Distribution of tandem repeats across chromosomes**. Rice chromosome 1 (top/left) Arabidopsis chromosome 3 (bottom/right). One or a maximum of two specific regions have significantly higher repeats near the centromere position.

**Figure 3:**
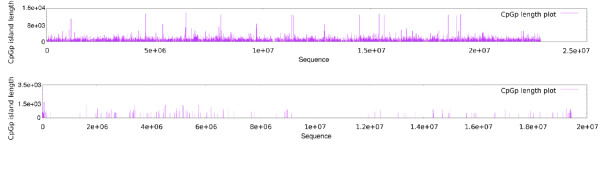
**CpG Islands**. Comparison of rice and Arabidopsis CpG islands across Rice chromosome 10 on top, and Arabidopsis chromosome 2 on the bottom. CpG islands are more frequent in Rice chromosomes.

Figure 2 shows the distribution of tandem repeats along the chromosomes 1 and 3 of rice and Arabidopsis, respectively. The graphs indicate specific regions in chromosomes with significantly higher repeat's length. CpG island graphs do not present any particular pattern by their own, but comparing rice and Arabidopsis chromosomes (here; chromosome 10 and 2, respectively) it seems that rice chromosomes include many more CpG islands than Arabidopsis (Figure 3).

### Comparison of curvature and CpG islands

**Figure 4:**
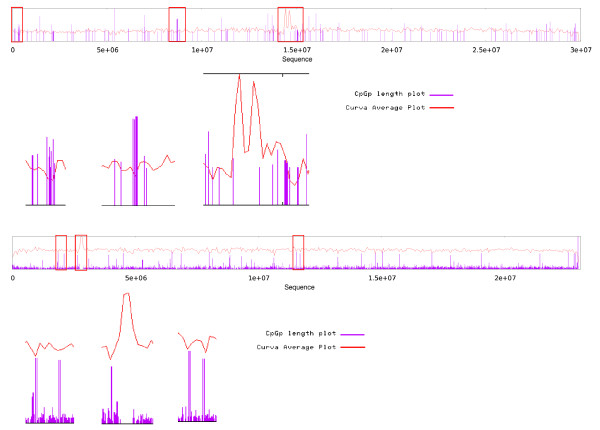
**Joint plot of curvature and CpG islands**. Most CpG islands occur in regions with minimal curvature values. In centromeric regions, where curvature usually has its highest value, CpG islands disappear. The plot shows Arabidopsis chromosome 1 (top) and rice chromosome 9 (bottom).

Figure 4 shows a combined-plot of curvature and CpG islands for Arabidopsis chromosome 1 and rice chromosome 9. The plot shows a clear relationship between curvature and CpG islands, since most CpG islands occur in regions with minimal curvature value (MinCV). This behavior is consistently present in the majority of chromosomes of both genomes and concurrently in centromeric regions, where curvature usually has its highest value and where CpG islands are scarce. It has been shown that GC content impacts the structure of the DNA molecule and that curved regions tend to appear in GC poor regions [5], but GC content is not sufficient to determine the curvature profile of a DNA molecule. Since the curvature depends on the cumulative effects of the sequence in a long DNA region, it is possible to obtain two DNA fragments with exactly the same GC content but completely different curvature profiles, depending on the order on which the nucleotides appear. The evaluation of both CpG islands and curvature profile of second and third order Markov-chain permutations of chromosomes from Arabidopsis and Rice showed that the presence of both CpG islands and highly curved regions are non -random events that depend directly on the sequence order (see additional file 2 "Markov-plots", figures S1 and S2).

### Outlook of curvature and tandem repeats

**Figure 5:**
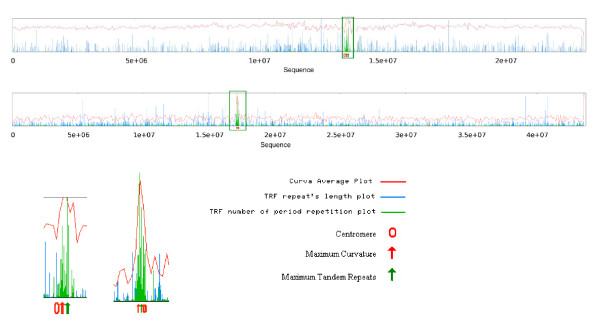
**Joint plot of curvature and repeats**. Regions with maximal repeats value locate exactly in centromeric regions, inside or adjacent to regions with maximum curvature. The plot shows Arabidopsis chromosome 3 (top/left) and rice chromosome 1 (bottom/right).

**Figure 6:**
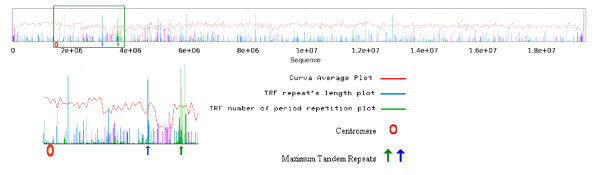
**Exceptional chromosome plot**. In this example plot of Arabidopsis chromosome 2, minimal curvature locates exactly between two maximal tandem repeat regions; inside the low curvature region, tandem repeats are scarcely seen. This chromosome shows no coincidence between the tandem repeat regions, curvature maximal values and the centromere position.

One of the major features revealed by combined plots of Arabidopsis chromosomes 1, 3 and 4 and Rice chromosomes 1 to 6, 8, 9, and 12 is that regions with maximal repeat values are located exactly in the centromeric regions and inside or adjacent to the regions with MaxCV. Figure 5 shows the relationship between curvature and tandem repeats for Arabidopsis chromosome 3 and rice chromosome 1. Chromosome 2 of Arabidopsis (Figure 6) and chromosomes 10 and 11 of rice (data not shown) do not follow any of these two patterns, instead these chromosomes show a significant MinCV, and surprisingly it is located between two maximal tandem repeats. Tandem repeats are barely seen next to or inside minimal curvature regions. Also, as a general observation, as the repeat number increases, the value of curvature increases in both Arabidopsis and rice chromosomes 1, where in a plot of repeat number versus curvature, a cluster of high curvature points in the case of 100 or more repeats in a 20 kb window is clearly observed (see additional file 3 "repeat-plots", figure S3). Further studies of this chromosome's peculiar structure might shed light upon their origins and evolutionary history.

### Relationship between CpG Islands and Tandem Repeats

**Figure 7:**
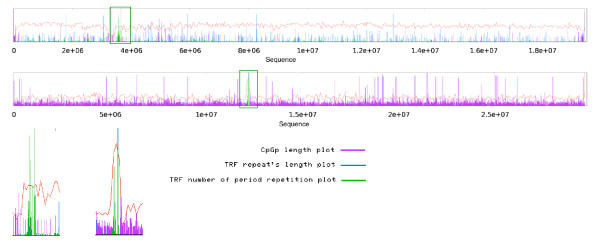
**CpG islands and repeats**. In these example plots of Arabidopsis chromosome 2 (top/left), and rice chromosome 7 (bottom/right) the regions with highest repeat length coincide with low or no CpG presence.

Analysis of the location of CpG islands with the distribution of tandem repeats across the chromosomes showed that regions enriched with repeats usually do not contain any CpG islands, but the opposite situation does not always happen, in which regions with few CpG islands do not necessarily have more repeats (Figure 7). In this case, as a general observation, when repeat number increases, the total length of CpG islands decreases. The plot of repeat number versus CpG length shows a lower length of CpG islands in the case of 100 or more repeats in a 20 kb window in comparison to repeats of 1 to 99 (see additional file 3 "repeat-plots", figure S3).

### The exceptional chromosomes

Chromosomes 2 and 5 in Arabidopsis and chromosomes 10 and 11 in rice show different patterns for all the features analyzed (Figure 1 andTable 2). This might indicate a different evolutionary history for these chromosomes, but the specific reasons for their exceptional characteristics need to be further elucidated. A wider survey of different organisms, as their genomic sequences become available, might provide the necessary data to elucidate if these chromosomes' structure is present or conserved in other kingdoms.

**Table 2:** Statistical values of DNA curvature in Arabidopsis (Ath) and rice (Osa) chromosomes.

Chromosome	Curvature avg.	SD	Curvature max.	z-score
Ath_chr01	0.222	0.007	0.291	9.477
Ath_chr02	0.222	0.007	-	-
Ath_chr03	0.221	0.007	0.246	3.792
Ath_chr04	0.221	0.007	0.259	5.739
Ath_chr05	0.222	0.006	0.246	4.324
				
Osa_chr01	0.201	0.007	0.261	8.464
Osa_chr02	0.202	0.007	0.269	9.580
Osa_chr03	0.201	0.006	0.224	3.932
Osa_chr04	0.199	0.007	0.230	4.071
Osa_chr05	0.201	0.008	0.230	3.841
Osa_chr06	0.201	0.008	0.252	6.656
Osa_chr07	0.202	0.008	0.254	6.515
Osa_chr08	0.202	0.008	0.233	4.124
Osa_chr09	0.202	0.010	0.279	7.400
Osa_chr10	0.201	0.008	-	-
Osa_chr11	0.203	0.007	-	-
Osa_chr12	0.203	0.008	0.241	4.893

Whole chromosome curvature averages are shown, with the corresponding Standard Deviation (SD). Curvature max. indicates the highest curvature value found in each chromosome, for the cases where this value represented more than 3 Standard Deviation Units (SDU) from the corresponding mean. The z-score measures the distance in SDU from the mean to the maximal value. Chromosome 2 of Arabidopsis and chromosomes 10 and 11 of rice do not present any curvature value above the described threshold.

### Comparison with other organisms

Whole chromosome sequence data from the genomes of *Mus musculus *(mouse) and *Saccharomyces cereviciae *(yeast) was analyzed to establish if the structural features we describe here were conserved beyond the plant kingdom. None of the chromosomes of these organisms presented a similar structure near/on the centromeric region. It is worth noting that the mouse centromeres are telocentric, and so might have different structural requirements than the submetacentric centromeres in plants, but in the case of yeast, which also presents submetacentric centromeres, there is no indication of repeat regions or high curvature near the centromeres similar to the profiles found in plant chromosomes. It is important to note that previous reports of DNA curvature in the centromeres of yeast [6] studied only fragments of 300 nucleotides for the calculations, describing a very localized feature; a bend in the middle of a DNA fragment of 110 base pairs containing CEN fragments. Such features are not detected by our approach as the use of the smoothing algorithm averages local variations in curvature to favor the identification of larger, more global features.

The negative relationship between CpG islands and curvature was evident also in mouse chromosomes, but not in yeast, where both CpG islands and repeat regions are very scarce. Multiple plots of DNA curvature, repeats and CpG islands for all chromosomes of the aforementioned organisms are available in additional file 4 "yeast-mouse-plots".

## Conclusion

This study presents a systematic genome-scale analysis of DNA curvature, CpG islands and tandem repeats at the DNA sequence level in rice and Arabidopsis. It reveals significant correlations between curvature and genomic features such as CpG islands and repeat distribution. The detailed analysis of each feature and the results driven from the combined plots generally propose that, for most of the chromosomes, maximal DNA curvature occurs adjacent to centromeric regions, which also happen to have high frequency of repeats (only tandem repeats in this study). In rice, it has been shown that the centromere is occupied by a centromere-specific retrotransposon [29]. There is also a negative correlation between CpG islands and DNA curvature value along the chromosomes and centromeric regions, where usually maximal curvature regions are free of CpG islands. Previous studies have shown correlations between AT-content and curvature, which demonstrated that high AT-content might be responsible for the high curvature values [30]. Although, later studies [31,32] recall the question of the evolutionary constraints acting on these sequences and whether we should expect that DNA curvature can result from sequence elements other than AT tracts.

Our results suggest a genome evolution scenario in which an increase in tandem repeats, both in length and repetition increases the DNA curvature, which in turn decreases GC content and subsequently promotes loss of CpG islands. Maximal curvature usually occurs at centromeric regions, as it has been already suggested by previous studies in other organisms, which have shown similar features [6,7]. Here we extend these previous observations by describing these structural features in all complete chromosomes of two plant genomes and finding correlations between repeats, curvature and the centromere. The most critical finding or question remaining in this work is that in contrast to other prokaryote and eukaryotes studied before, in plants some chromosomes (chromosomes 2 and 5 in Arabidopsis and 10 and 11 in rice), do not follow the same pattern or rules for structural features such as curvature value, CpG islands or distribution of tandem repeats. This shows the need for further research at both experimental and computational levels to explain this discrepancy.

## Methods

The source data were the Arabidopsis and rice complete genome sequences in XML format downloaded from **TAIR **http://www.arabidopsis.org/ and **International Rice Genome Sequencing Project **http://rgp.dna.affrc.go.jp/IRGSP/, respectively. The genomic sequences for each chromosome were extracted from the XML files and stored in FASTA format. Sequences were filtered by masking any characters not present in the set S = {A, C, G, T, a, c, g, t}.

In order to compare plants with other organisms, sequence data from *Mus musculus *and *Saccharomyces cereviciae *was obtained from the **NCBI FTP site **ftp://ftp.ncbi.nih.gov/genomes/. Centromere positions were collected from the **Saccharomyces Genome Database **http://www.yeastgenome.org/ and the **UCSC Genome Browser **http://genome.ucsc.edu/.

### Genome-scale curvature calculation

The computation of the distribution of curvature of DNA sequences was performed using the CURVATURE program [5]. This program calculates the three-dimensional path of DNA molecules and estimates the segment curvature by computing the radius of the arc approximating to the path of the axis of the DNA fragment. The dinucleotide wedge angles of Bolshoy [4] and the twist angles of Kabsch [33] were used for all calculations. Whole chromosome sequences were used as input and maps of the curvature distribution using a window size of 125 bp along the whole sequence were produced. The DNA curvature was measured in DNA curvature units (cu) introduced by Trifonov and Ulanovsky [34] and used in all of the analyses. The scale of these "curvature units" ranges from 0 (e.g. no curvature) to 1.0, which corresponds to the curvature of DNA when wrapped around the nucleosome. For example, a segment of 125 bp of length with a shape close to a half-circle has a curvature value of about 0.34 cu. Such strongly curved regions with values of >0.3 cu appear infrequently in genomic sequences. Since each chromosome presents a specific curvature distribution, its average and standard deviation (SD) values can be used to define thresholds and identify significant features. In this study a curvature signal was identified as significant if a maximal curvature value was at least 3 SD above the genomic average. The output spatial mapping file consists of two columns; the first column enumerates bases (corresponding to length of the chromosome in base pairs) and in the second column, a floating-point number less than one (<1), represents the curvature value at each base pair along the chromosome. Since this "map" file, is too large to be plotted directly (the map file of chromosome 1 of rice, for example, has about 43 million curvature values), low perturbations were removed and high perturbations were emphasized. In order to attain this, we used a method that summarizes the curvature signal as described by the following algorithm.

### Signal Processing

**Figure 8:**
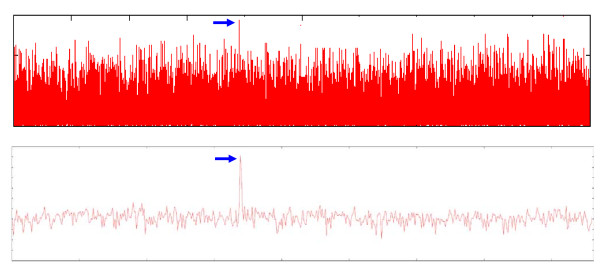
**Chromosomal curvature signal**. Signal of the curvature value before (top) and after (bottom) applying the signal processing algorithm. Locations of maximal curvature values are marked by a blue arrow.

Our method considers a sliding window on a given signal that covers only part of the signal and each window contains a signal fragment with some high and low perturbations. In each window, we determine extreme points by a simple analysis in *O(n) *time complexity. When each point has a bigger or lower value than both its predecessor and successor points, it is called a maximal or minimal point and collected as an apex value. Thereafter in each window, two base lines for positive and negative apex values are defined such that via these base lines we construct two new coordinates for the signal's peak values. These new coordinates are suitable for exaggerating low and high perturbations. To describe this method, we focused first on positive values; if the positive peaks' values are members of the set S_p_={P_1_, P_2_, ..., P_n_}, the mean value (M_p_) of the set can show the base line of positive apexes. By using M_p_, a new set of positive apexes can be reached by subtracting M_p_; thus giving a new_S_p_={ P_1_-M_p_, P_2_-M_p_, ..., P_n_-M_p_}. Here the application of an exponential function {e^x^| x is member of new_S_p_} will emphasis high apex values and reduce low apex values. This process of changing coordinates is a type of kernel function, as used on statistical machine learning approaches (such as support vector machines). Through this change, the system's low perturbations, which have negative values in our exponential function, will be projected into small values whereas high perturbations that have positive values will be mapped to exponentially higher values after performing the exponential function. The process of analyzing negative apex values S_n_= {N_1_, N_2_, ..., N_m_} is similar to the positive values where the exponential function has changed to {-e^-x^| x is member of new_S_n_}. The details of the algorithm are presented below. Figure 8 shows the curvature signals before and after applying the algorithm.

### Algorithm for Signal Processing

//For a given signal S with L sample points in an array S[1...L]

Begin

Tentative window length = L/5

For *j*:0 to 5 do

   //Determining maximal and minimal points

   For *i*:j*L/5 to (*j*+1)L/5 do

      If (*S*[*i*] is a positive apex)

         Add *i *to *S*_p_

      If (*S*[*i*] is a negative apex)

         Add *i *to *S*_n_

   //Computing mean values

   

   

   //Changing coordinates

   For *i*:1 to *n *do

      

   For *i*:1 to *m *do

      

   //Performing exponential functions

   For *i*:1 to *n *do

      

   For *i*:1 to *m *do

      

End

### Computation of tandem repeats and CpG islands

Tandem repeats across whole chromosomes were first detected using the Tandem Repeats Finder (TRF) program version 4.0 [35]. Tandem Repeats Finder is an application for finding tandem repeats in DNA sequences, that employs a stochastic model of repeats and associated statistical detection criteria. We scanned CpG Islands in genomic sequences using the Takai and Jones algorithm [26], which is optimized for searching CpG Islands (CGI) in whole genomes. Its search criteria are GC content ≥ 55%, ObsCpG/ExpCpG≥0.65, and length ≥ 500 bp. Based on this algorithm, we used eight iterative steps to scan all the possible CGI in each genome as follows: (1) Set a window size of 125 bases at the start position of a sequence and calculate GC content (%) and ObsCpG/ExpCpG in the first window. Here, ObsCpG/ExpCpG = NCpG/(NC × NG) × N where NCpG, NC, NG, and N are, respectively, the number of dinucleotide CpGs, nucleotide Cs, nucleotide Gs, and all nucleotides (A, C, G, and T) in the sequence (i.e., 0 nucleotides). Shift the window 1 base each time until the window meets the criteria for a CGI. (2) Once a seed window (i.e., it meets the criteria) is found, move the window 150 bases forward and then evaluate the new window again. (3) Repeat step 2 until the window does not meet the criteria. (4) Shift the last window in steps of 1 base each time towards the 5' end until it meets the criteria again. (5) Evaluate the whole segment (i.e., from the start position of the seed window to the end position of the current window). If it does not meet the criteria, trim 1 base from each side until it meets the criteria. (6) Connect two individual CGI fragments if less than 100 bases separate them. (7) Repeat step 5 to evaluate the new sequence segment until it meets the criteria. (8) Reset start position immediately after the CGI identified at step 7 and go to step 1.

### Statistical analysis

The statistical significance of the features described above was calculated by measuring the average and distribution of curvature along the genome, as well as CpG and repeat numbers in non-overlapping windows along the genome. These distributions were used to calculate the SD, and significant features were selected by setting a threshold on the value corresponding to 3 SD. Features with values above this threshold were collected as significant (Table 2). The z-score, calculated by subtracting the average from the peak value, and dividing by the SD, gives a measure of the statistical distance between the observed feature and the natural average, and can be expressed as a probability.

A modified Markov-chain permutation process was used to obtain permuted chromosomes that conserve dinucleotide and trinucleotide distributions; the chromosome DNA sequence was split into all dimers (in 2 phases) and all trimers (in 3 phases), and the set of dinucleotides or trinucleotides was shuffled. The permuted chromosomes obtained in this manner were subjected to the same analysis as the natural chromosomes. No statistically significant features were identifiable in these permuted cases. In the additional file 2 "Markov-plots", figure S1 presents an overlay of curvature plots for a natural and trinucleotide-permuted chromosome.

### Integrated plotting of the curvature, repeats and CpG islands

Integrated results of the three analyses mentioned above for the 12 and 5 chromosomes of rice and Arabidopsis, respectively, were drawn in individual and combined-plots, using the freely distributed **Gnuplot **program http://www.gnuplot.info/ in individual and mixed plots based on different parameters. Perl scripts for extracting proper data from source result files and generating plots were developed in-house. Gnuplot parameters were automatically set and final plots saved in png format. The source code of all Perl scripts is freely available upon request.

## Authors' contributions

AM devised the experiment and drafted the manuscript. SM developed the software and analyzed the data. RJ developed the software and drafted the manuscript. All authors read and approved the manuscript.

## Supplementary Material

Additional file 1 **Plots showing curvature**. CpG and repeats for all chromosomes of Arabidopsis and rice. Click here for file

Additional file 2 **Plots showing curvature, CpG and repeats for the Markov-permutations for Arabidopsis and rice first chromosomes**. Click here for file

Additional file 3 **Plots showing curvature**. CpG and repeats for all chromosomes of yeast and mouse. Click here for file

Additional file 4 **Plots showing repeat number vs. curvature average and CpG length in 20 kilobase windows for Arabidopsis and rice first chromosomes**. Click here for file
