# Supplementary material for: Genome-scale computational analysis of DNA curvature and repeats in Arabidopsis and rice uncovers plant-specific genomic properties
Source: BMC Genomics. 2011 May 6;12:214. doi: 10.1186/1471-2164-12-214 (PMC3113785; doi:10.1186/1471-2164-12-214)

Multiple plots with curvature profile (red line), CpG islands (purple lines) and repeats (green and blue lines) for all chromosomes from Arabidopsis (ath) and rice (osa)

ath01


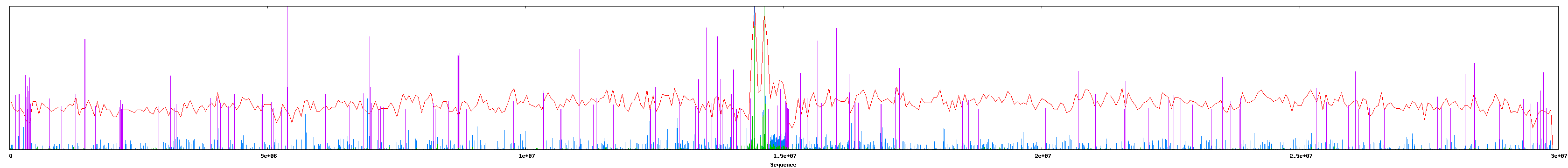


ath02


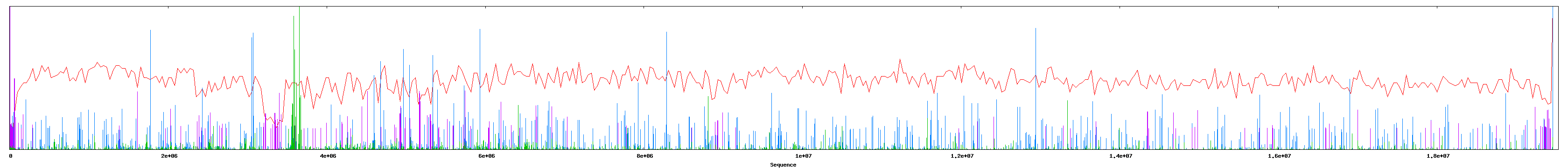


ath03


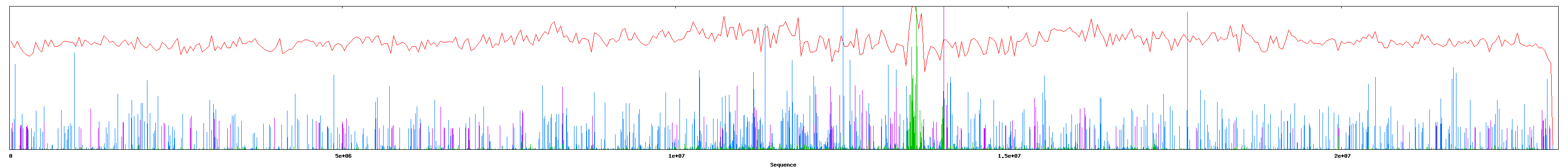


ath04


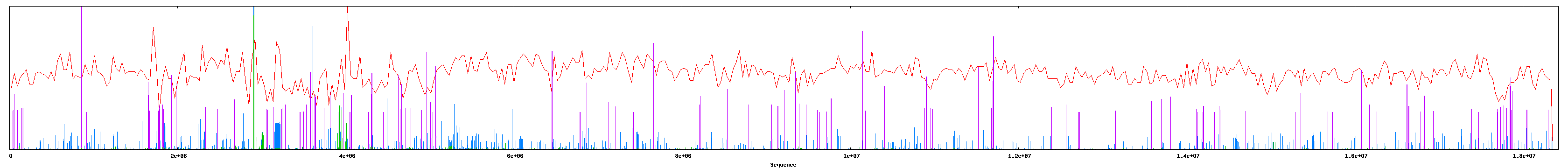


ath05


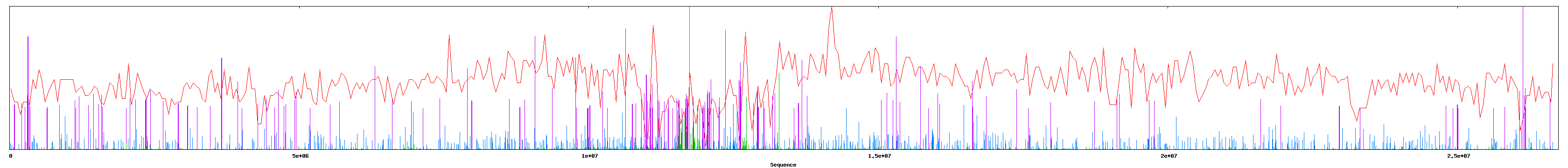


osa01


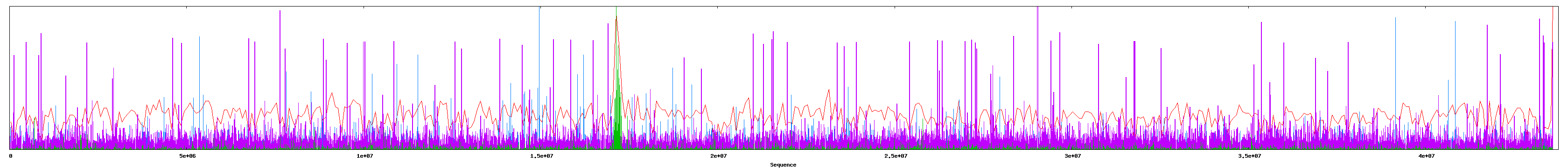


osa02


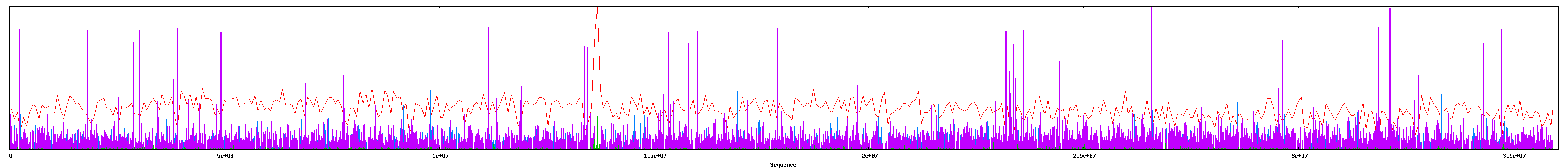


osa03


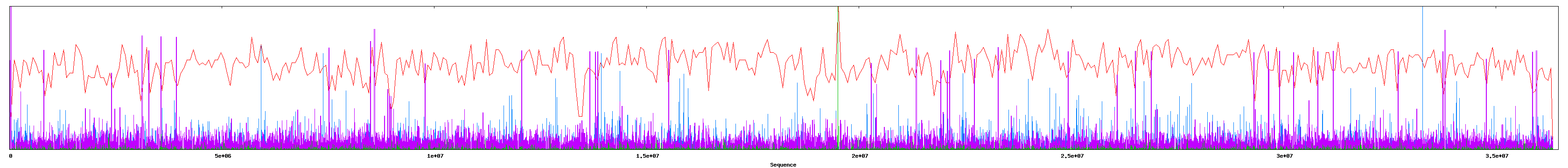


osa04


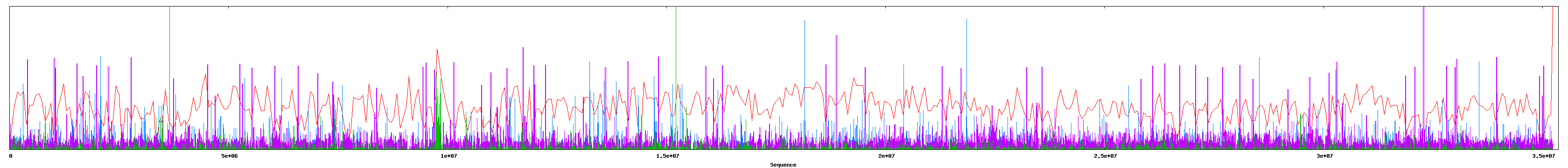


osa05


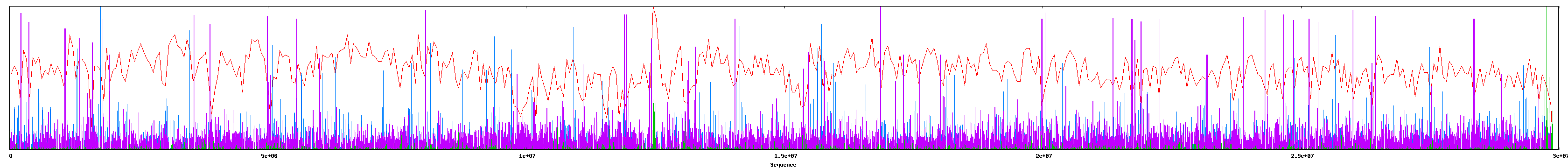


osa06


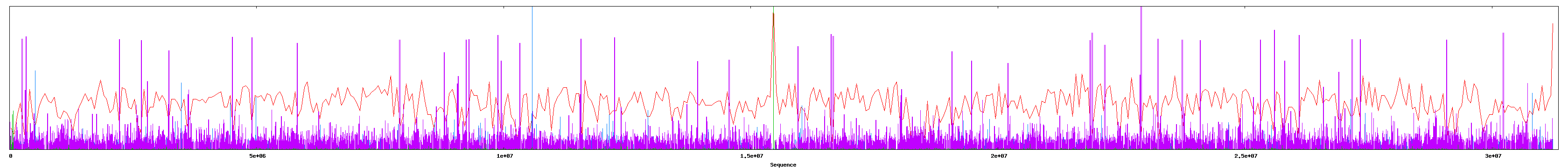


osa07


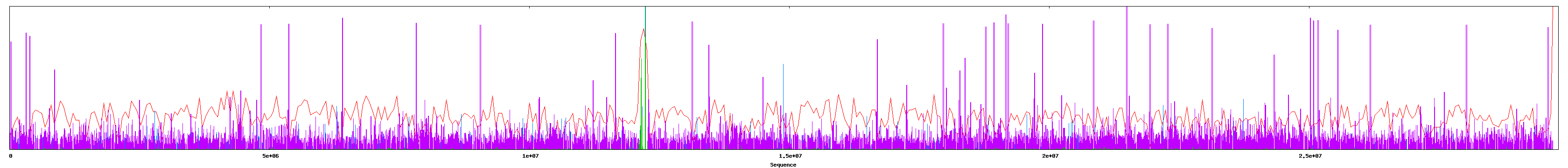


osa08


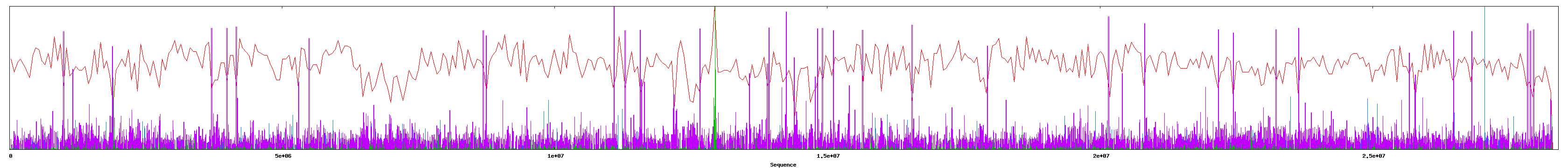


osa09


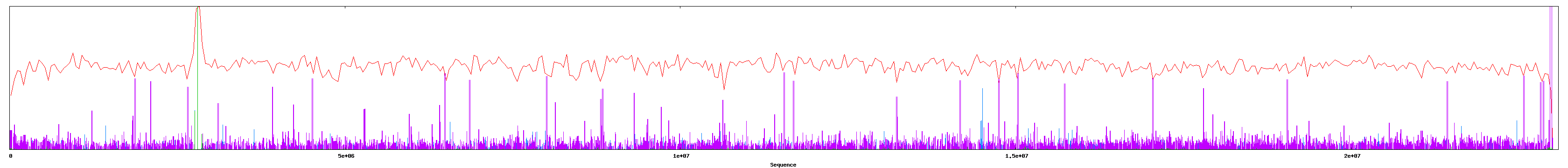


osa10


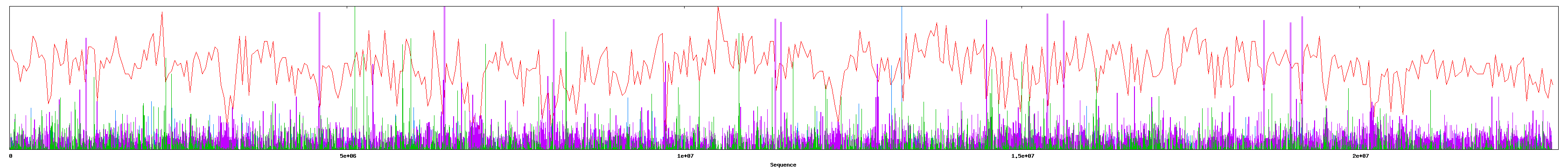


osa11


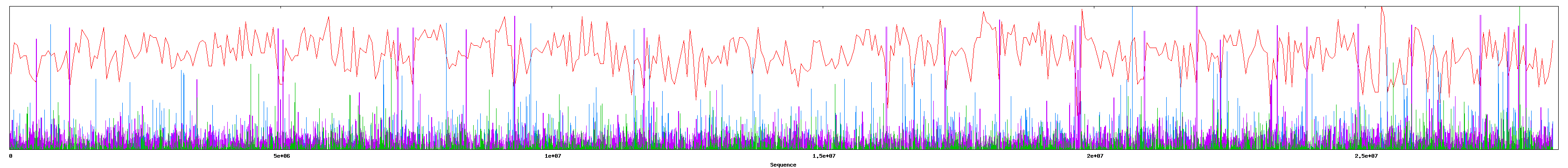


osa12


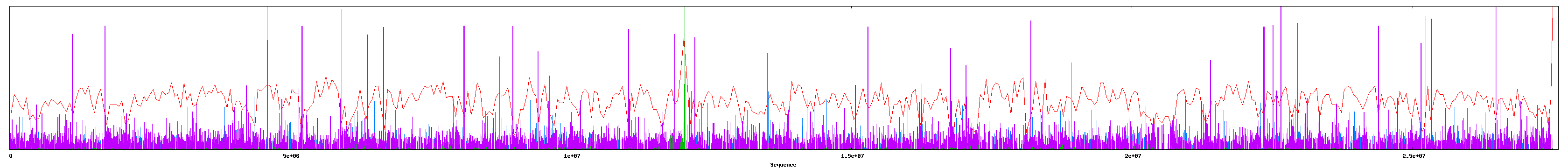

Supplement: Additional file 1 — Plots showing curvature. CpG and repeats for all chromosomes of Arabidopsis and rice. [file 1471-2164-12-214-S1.DOC]
