# Supplementary material for: Genome-scale computational analysis of DNA curvature and repeats in Arabidopsis and rice uncovers plant-specific genomic properties
Source: BMC Genomics. 2011 May 6;12:214. doi: 10.1186/1471-2164-12-214 (PMC3113785; doi:10.1186/1471-2164-12-214)
Supplement: Additional file 2 — Plots showing curvature, CpG and repeats for the Markov-permutations for Arabidopsis and rice first chromosomes. [file 1471-2164-12-214-S2.DOC]

Markov-chain permutations of the first chromosomes from Arabidopsis and rice.


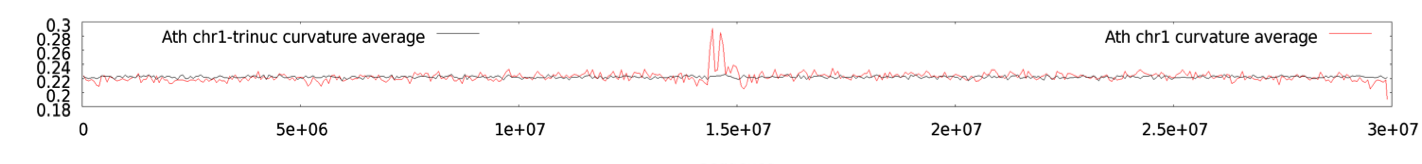


Figure S1. Curvature profiles of trinucleotide-based permutation of chromosome 1 from Arabidopsis (gray line), together with the natural chromosome (red line). The curvature signals characteristic of the centromere are missing in the permuted version.


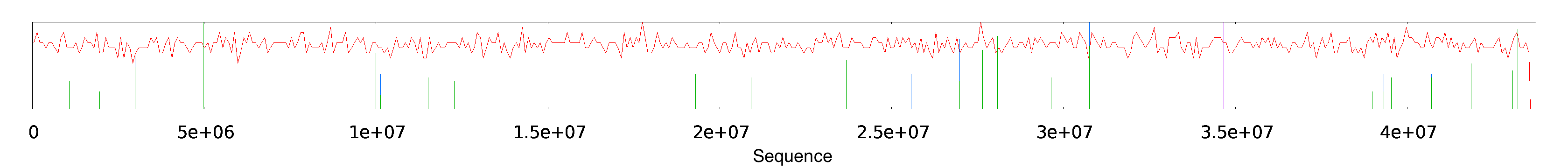
Figure S2. Curvature profile (red line), CpG islands (purple lines) and repeats (green and blue lines) found in the trinucleotide-based permutation of rice chromosome 1. All characteristic features of this chromosome are missing in the permuted version.
