# Supplementary material for: Genome-scale computational analysis of DNA curvature and repeats in Arabidopsis and rice uncovers plant-specific genomic properties
Source: BMC Genomics. 2011 May 6;12:214. doi: 10.1186/1471-2164-12-214 (PMC3113785; doi:10.1186/1471-2164-12-214)

Multiple plots with curvature profile (red line), CpG islands (purple lines) and repeats (blue and green lines) for all chromosomes of *Mus musculus* (mouse, mmus) and *Saccharomyces cereviciae* (yeast, scer).

Mouse -- mmus01


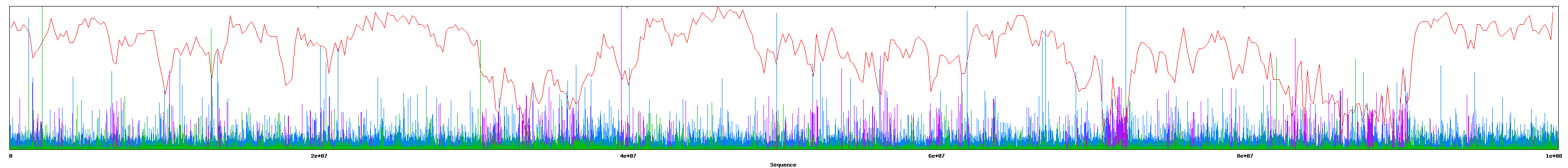


mmus02


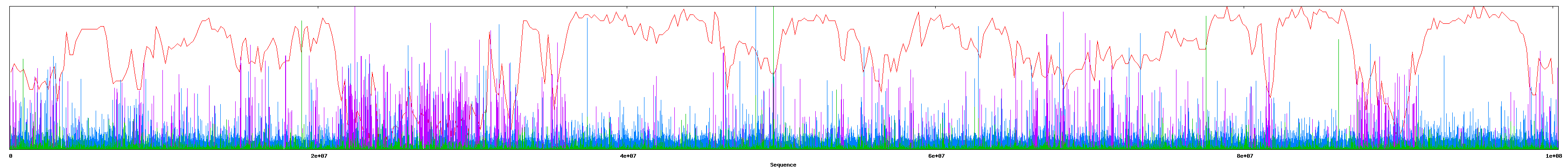


mmus03


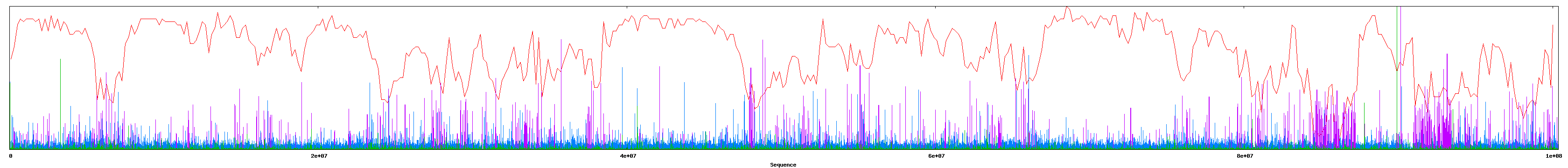


mmus04


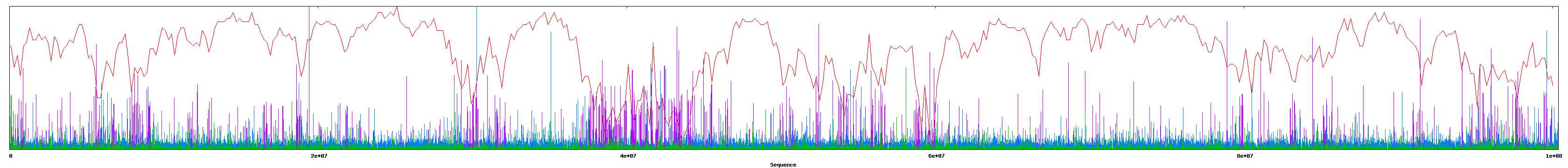


mmus05


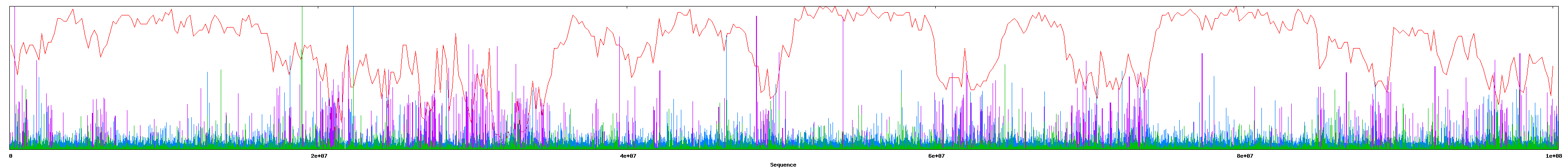


mmus06


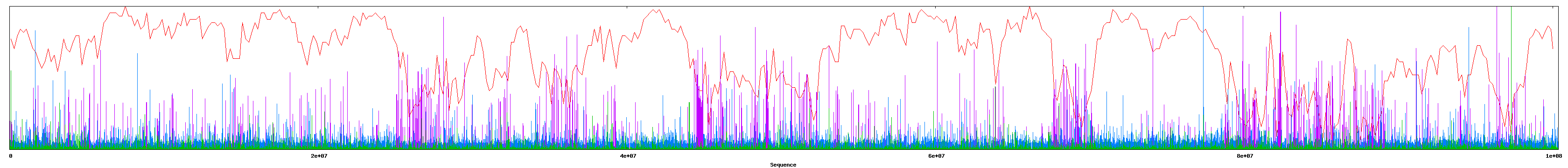


mmus07


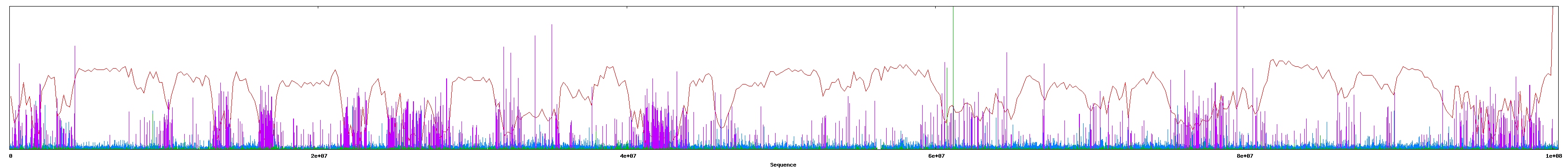


mmus08


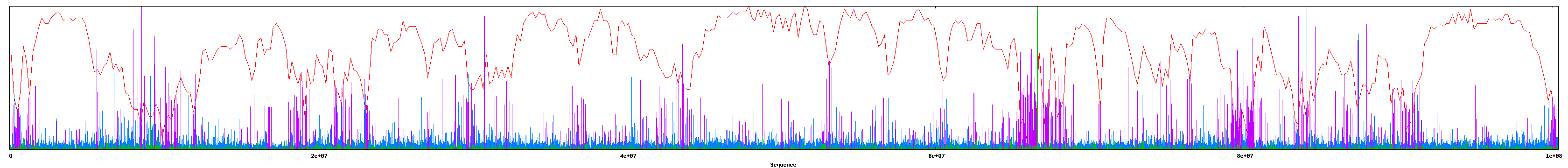


mmus09


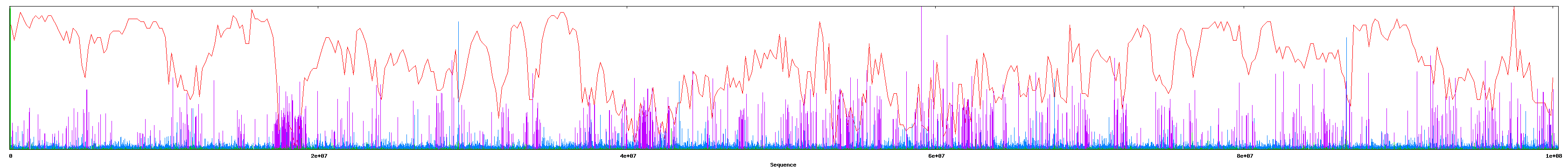


mmus10


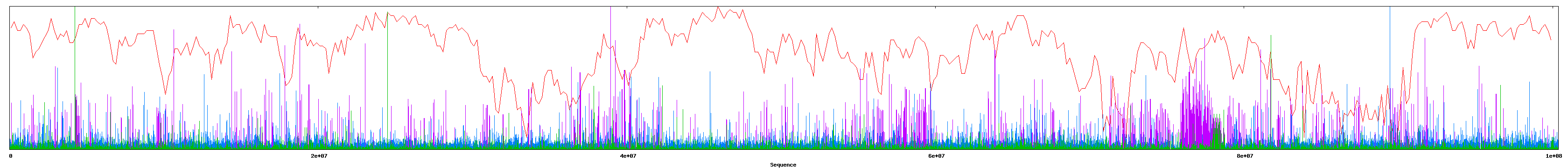


mmus11


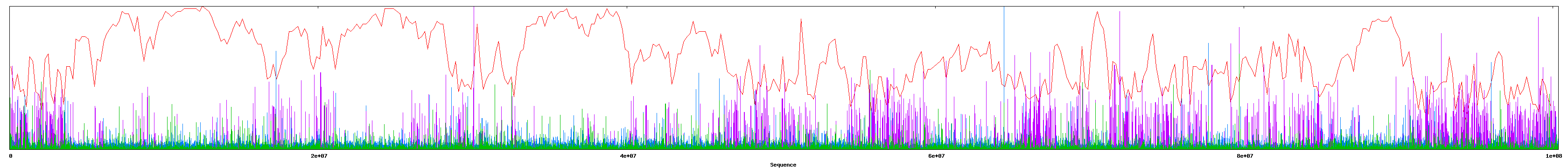


mmus12


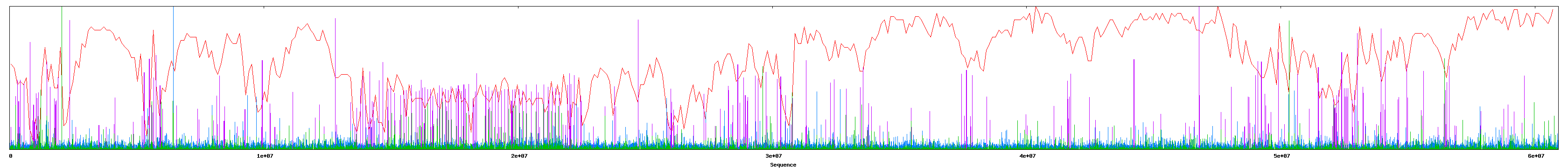


mmus13


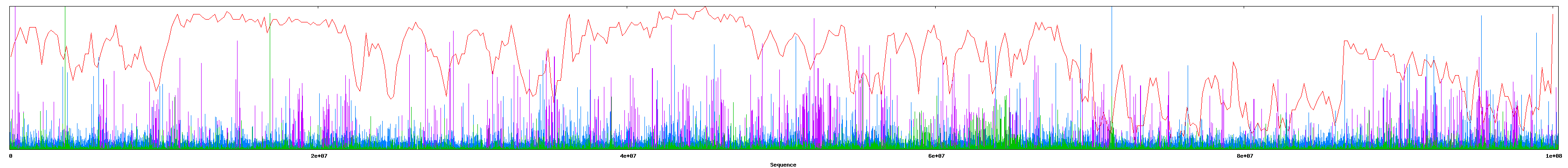


mmus14


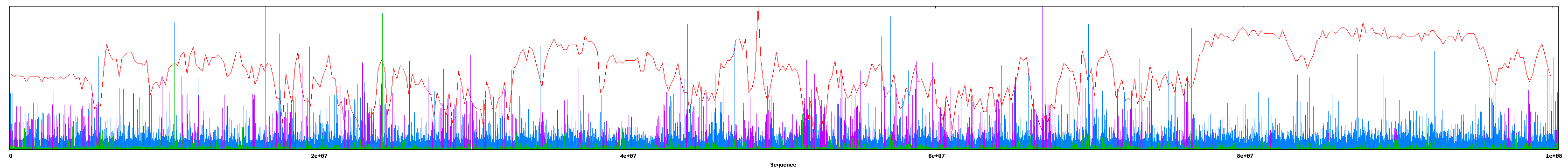


mmus15


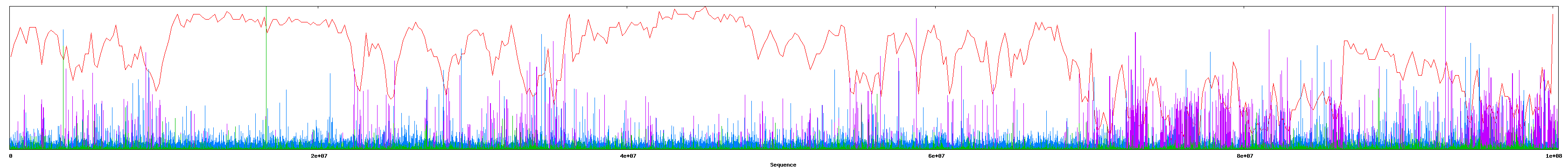


mmus16


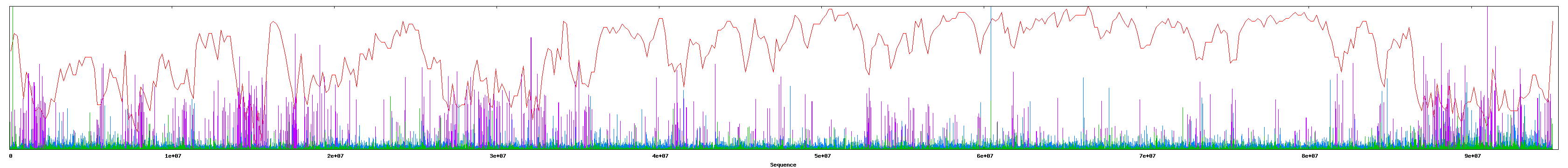


mmus17


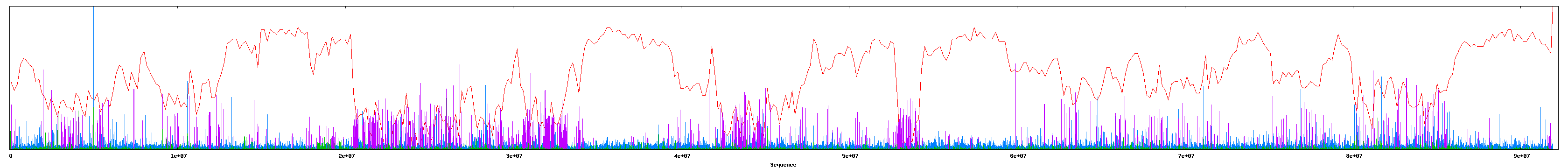


Mmus18


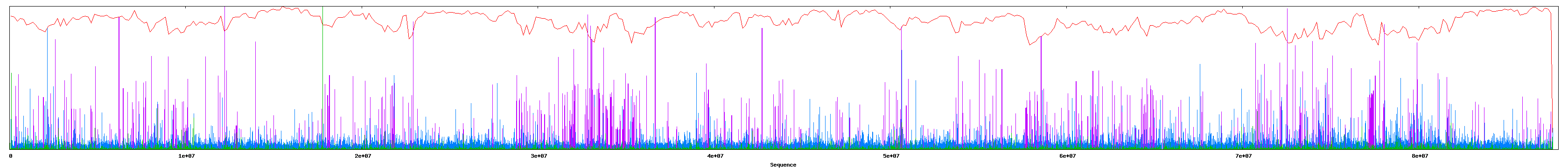


mmus19


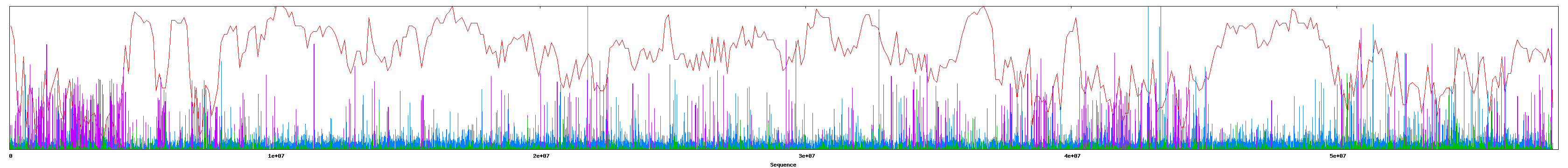


mmusX


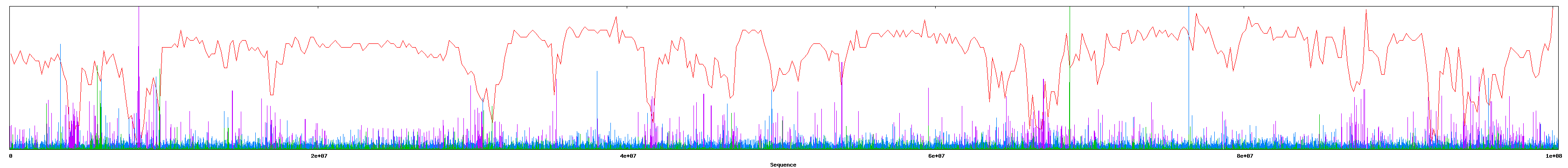


mmusY


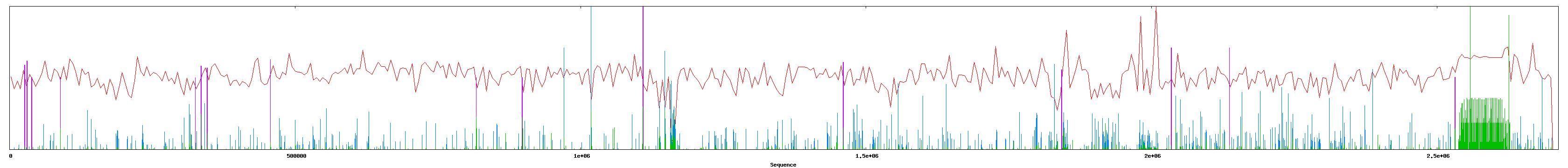


Yeast -- scer01


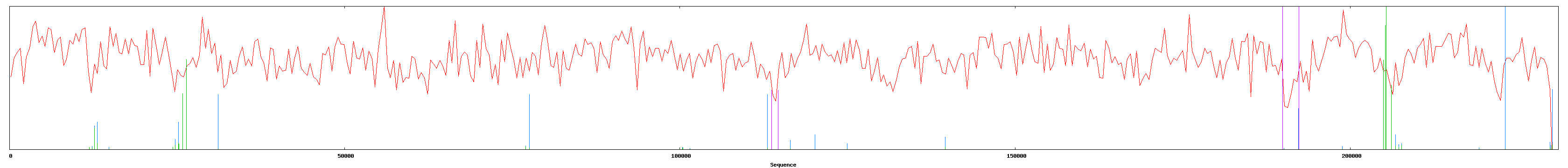


scer02


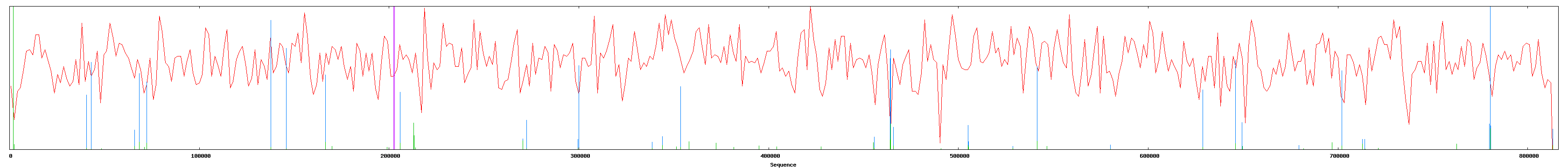


scer03


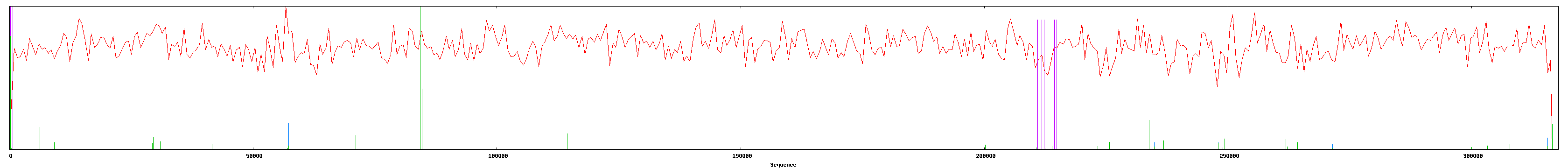


scer04


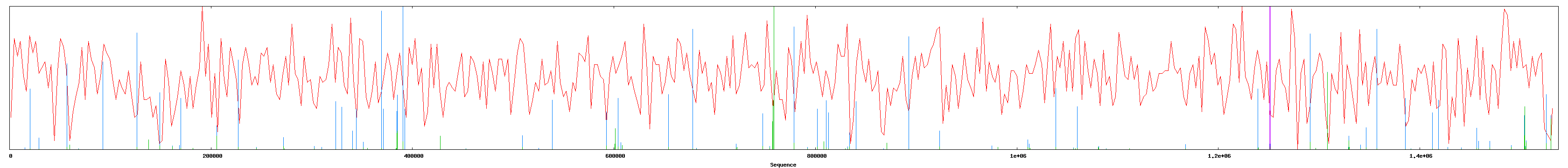


scer05


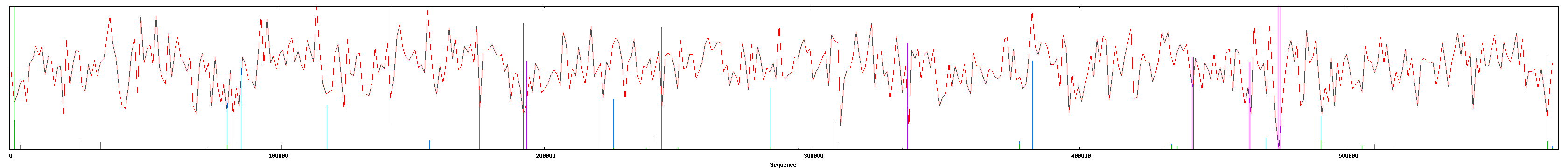


scer06


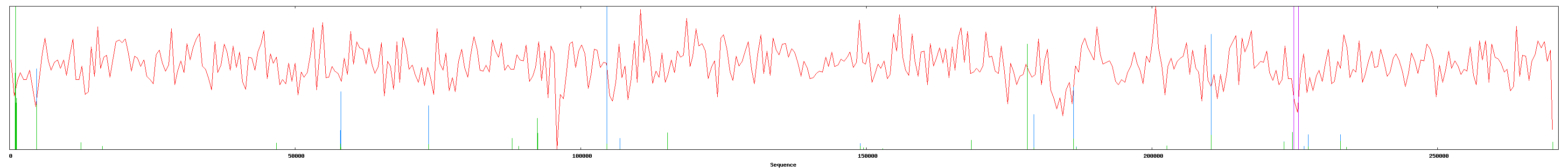


scer07


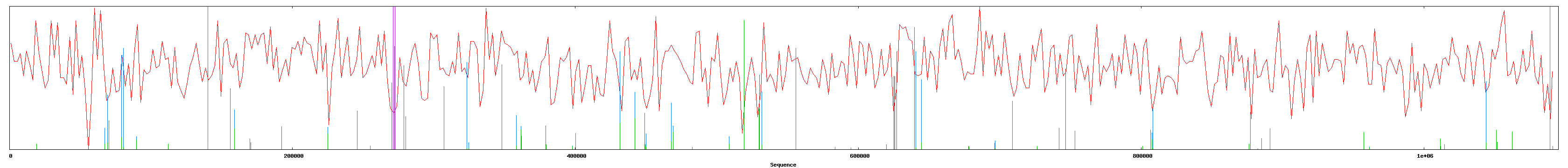


scer08


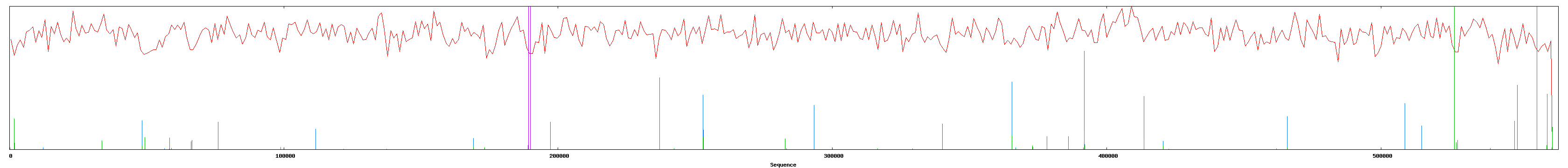


scer09


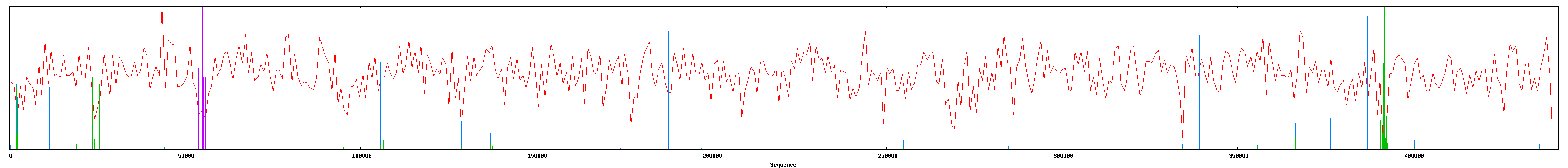


scer10


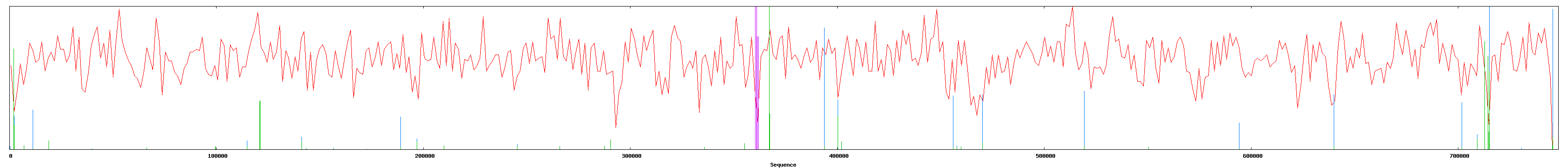


scer11


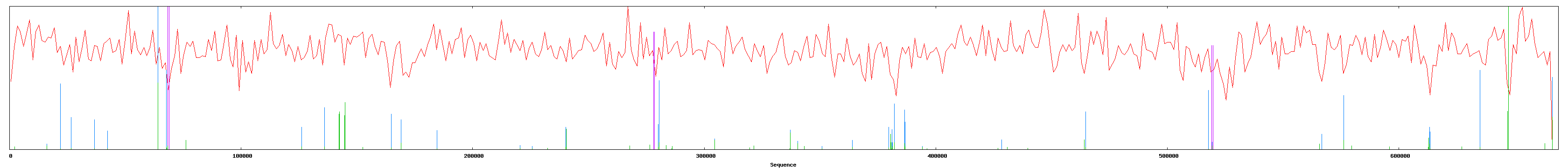


scer12


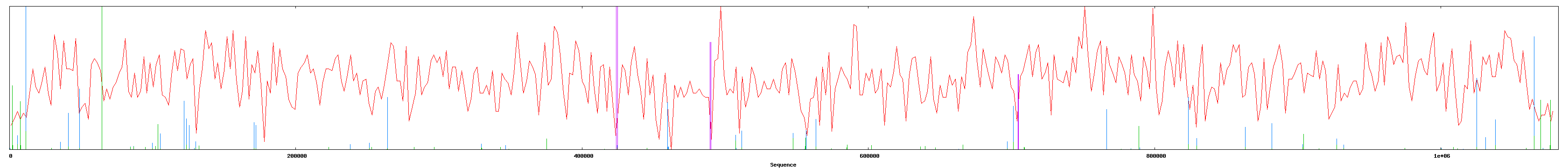


scer13


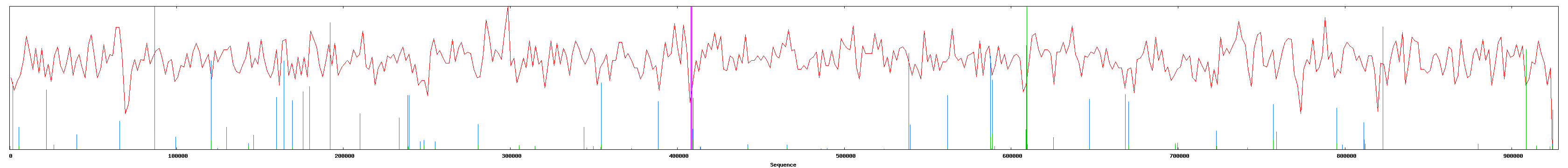


scer14


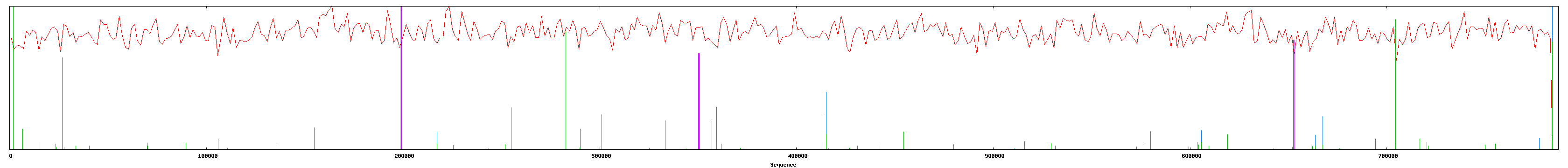


scer15


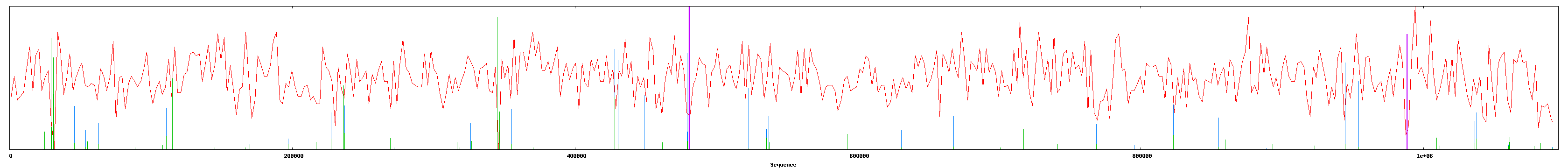


scer16


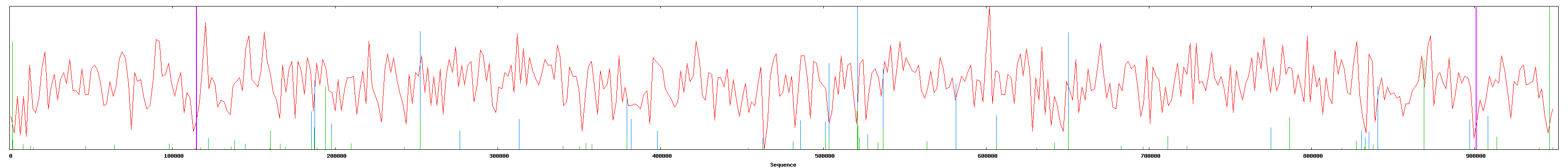

Supplement: Additional file 3 — Plots showing curvature. CpG and repeats for all chromosomes of yeast and mouse. [file 1471-2164-12-214-S3.DOC]
