# Supplementary material for: Genome-scale computational analysis of DNA curvature and repeats in Arabidopsis and rice uncovers plant-specific genomic properties
Source: BMC Genomics. 2011 May 6;12:214. doi: 10.1186/1471-2164-12-214 (PMC3113785; doi:10.1186/1471-2164-12-214)
Supplement: Additional file 4 — Plots showing repeat number vs. curvature average and CpG length in 20 kilobase windows for Arabidopsis and rice first chromosomes. [file 1471-2164-12-214-S4.DOC]

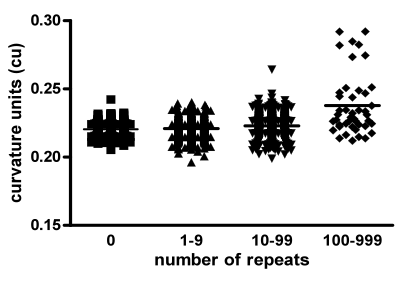

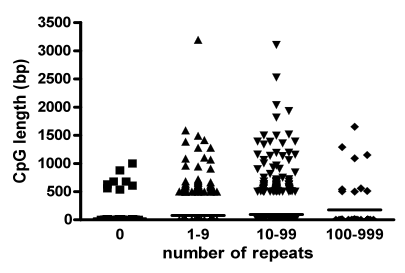


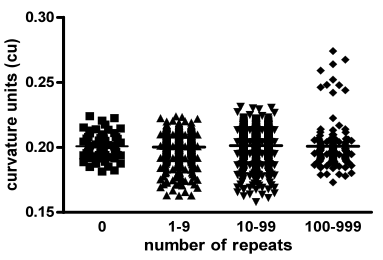

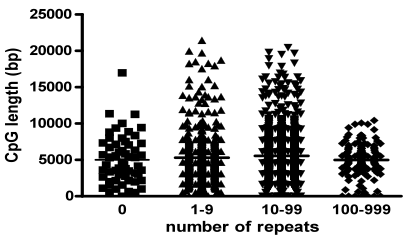


Figure S3. Repeat plots.

Plots of repeat number vs. curvature average and CpG length in 20 kb windows for Arabidopsis (top) and rice (bottom) first chromosomes.

Repeat numbers were collected in 4 bins according to the numbers presented in the x-axis. Curvature tables present curvature units (cu) and CpG islands total sequence length in the y-axis.
